# Modeling the distribution of soil organic carbon in salt marshes dominated by various plant species along Egypt’s Delta coast

**DOI:** 10.1186/s12870-026-09221-2

**Published:** 2026-06-17

**Authors:** Ebrahem M. Eid, Asmaa A. Shahawy, Yassin M. Al-Sodany, Mohamed M. El-Khalafy

**Affiliations:** Department of Botany, Faculty of Science, Kafrelsheikh University, Kafr El- Sheikh, 33516 Egypt

**Keywords:** Blue carbon, Coastal marshes, Mathematical models, Nile Delta, Validation indices, Vertical distribution

## Abstract

**Background:**

Understanding the vertical distribution and storage of soil organic carbon (SOC) in arid coastal salt marshes is essential for assessing their role in blue-carbon sequestration. This study examines SOC patterns in marshes dominated by *Arthrocnemum macrostachyum*, *Halocnemum strobilaceum*, and *Salicornia fruticosa*, as well as unvegetated areas. Using 200 soil cores (2,000 samples), we applied allometric, exponential, and sigmoid models to predict volumetric SOC density (SOC_*v*_; kg C/m³) and cumulative SOC stocks (SOC_*c*_; kg C/m²) across depth profiles.

**Results:**

SOC content showed an inverse exponential relationship with soil bulk density across vegetation types, consistent with patterns documented in other salt-marsh and coastal wetland systems. The allometric model provided the best SOC_*v*_ predictions for *A. macrostachyum*, whereas the sigmoid model performed best for *H. strobilaceum*, *S. fruticosa*, and unvegetated sites, based on mean normalized average error (MNAE), mean normalized bias (MNB), and residual mean squares (RMS). All three models accurately reproduced SOC_*c*_ for within-core depth extrapolation, with no significant differences between measured and predicted values within the validation dataset.

**Conclusions:**

This study provides an improved understanding of SOC dynamics in arid coastal salt marshes and demonstrates the utility of depth-based mathematical models for predicting SOC storage. These findings support local-scale efforts to evaluate carbon-sequestration potential, though broader spatial validation across heterogeneous landscapes remains necessary.

**Supplementary Information:**

The online version contains supplementary material available at 10.1186/s12870-026-09221-2.

## Background

Salt marshes are vegetated coastal ecosystems recognized as among the most productive on Earth [1]. These ecosystems play a crucial role in regulating atmospheric carbon dioxide (CO₂) levels and serve as efficient carbon sinks, storing approximately 25% of global soil carbon [2]. Their estimated carbon burial rate is around 210 g C/m²/yr [3]. Unlike most upland soils, salt marsh sediments continuously capture and store carbon through ongoing plant growth and subsequent burial processes [4–5]. Consequently, they are recognized as key blue carbon (referring to carbon stored within the soils and vegetation of salt marshes, mangroves, and seagrass ecosystems [6] habitats and are notable for their highly efficient carbon sequestration capacity [7]. The ability of salt marshes to store carbon is strongly influenced by the diversity and abundance of the plant species present [8]. Therefore, plant community composition can significantly affect the distribution and accumulation of soil organic carbon (SOC) within these distinctive coastal ecosystems [7].

Numerous studies conducted in humid temperate and tropical regions have examined carbon distribution in coastal salt marshes [3, 6–17]. However, in sub-humid and arid regions (where variations in rainfall, evapotranspiration, and soil characteristics may significantly influence carbon storage [18] field-based research remains limited [19]. This highlights the urgent need for comprehensive data collection and analysis of blue carbon in these underrepresented areas. Moreover, accurately measuring carbon stocks at the site level is essential for understanding spatial differences in blue carbon storage and for assessing future changes due to habitat conservation, degradation, loss, or restoration [6]. Acquiring this critical knowledge can therefore make a significant contribution to global strategies for combating climate change.

Soils represent the largest carbon reservoir within the terrestrial carbon cycle, containing nearly three times more carbon than vegetation and approximately twice as much as the atmosphere [20]. Accurately estimating SOC stocks is of significant interest to a wide range of stakeholders, including the Intergovernmental Panel on Climate Change [21]. However, estimates of SOC stocks in coastal salt marshes are often subject to considerable uncertainty, which can lead to under- or over-estimations [10]. Accurately quantifying and modeling the spatial distribution of SOC in salt marshes requires the development of models that incorporate soil depth [22]. These models enable the mathematical estimation of SOC by integrating data collected from various depths [7]. This approach also facilitates the prediction of SOC levels in unsampled layers, potentially reducing the need for extensive field sampling when models are well-designed [23]. Modeling SOC as a depth-dependent function is particularly useful when working with soil datasets that lack consistent sampling across all layers [24].

Selecting appropriate mathematical models and parameterization techniques is crucial for accurately simulating SOC distribution in salt marsh environments [7]. Nonlinear models are especially effective, as they better capture the complexity and variability of ecological data compared to linear models [23]. Different modeling approaches vary in their ability to represent the vertical structure of SOC, which often follows a nonlinear pattern due to factors such as organic matter decomposition, root biomass distribution, and sediment accumulation history [7, 25]. By accounting for these dynamics, nonlinear models offer a more realistic representation of SOC across diverse environmental conditions [26].

Salt marshes found along the deltaic coast of Egypt serve as essential ecological systems, contributing to biodiversity conservation, shoreline stability, and carbon sequestration [27]. These environments typically develop in low-elevation coastal zones influenced by tidal fluctuations and high salinity, conditions that give rise to characteristic plant communities dominated by halophytic species [28]. In Egypt, salt marsh vegetation represents the second most prominent type of plant cover and is widespread not only along saline coastal stretches but also within inland depressions and oasis regions [29]. Much of this vegetation consists of succulent halophytes such as *Arthrocnemum*, *Halocnemum*, *Salicornia*, and *Zygophyllum*. The majority of the halophytic flora in these habitats are excretive species (including *Aeluropus*, *Limoniastrum*, *Sporobolus*, and *Tamarix*) while a smaller number, such as *Juncus*, accumulate salts internally [29]. Salt marsh plants play a crucial ecological role by stabilizing sediments, filtering contaminants, and providing vital feeding and breeding grounds for migratory birds and aquatic organisms [28]. Despite their importance, these coastal marshes are under increasing pressure from human activities (including expanding urbanization and overgrazing) along with the broader impacts of climate change. As a result, many marshes are experiencing degradation and a notable loss of biodiversity [27].

To the best of the authors’ knowledge, this is the first study to model SOC distribution in Egypt’s salt marshes. The central research question guiding this work is: How effectively can mathematical models (specifically allometric, exponential, and sigmoid functions) predict the depth-wise distribution of SOC in salt marshes dominated by different plant species along Egypt’s deltaic coastline? Addressing this question, we hypothesize that the vertical distribution of both volumetric SOC density (SOC_*v*_; in kg C/m³) and cumulative SOC stocks (SOC_*c*_; in kg C/m²) will vary significantly depending on the dominant plant species.

The significance of this study derives from its examination of critical gaps in SOC modeling within arid coastal salt marsh ecosystems, which remain largely underrepresented in global datasets. By evaluating the influence of distinct plant species on SOC distribution, this research contributes essential new understanding to a neglected domain. Ultimately, the development of depth-resolved, species-specific SOC models offers refined insights into carbon dynamics in hypersaline soils and enhances predictive accuracy for future SOC evaluations, conservation decision-making, and habitat restoration planning.

## Methods

### Study area

The study area is situated in the northern portion of the Nile Delta, between 31°26′–31°34′ N and 30°31′–31°36′ E. This area is positioned between the Rosetta branch on the western side and the Damietta branch on the eastern side. Encompassing roughly 22,000 km², the Nile Delta represents one of the oldest agricultural regions globally [30]. It contains nearly 63% of Egypt’s cultivated land [31] and is home to about 41% of the country’s population [32]. With a population density estimated at around 1,360 inhabitants per km², it is recognized as one of the most densely settled agricultural landscapes worldwide [31]. The northern Mediterranean fringe of the Delta experiences an arid climate [33], featuring warm summers with temperatures typically between 20 and 30 °C and mild winters where temperatures generally remain above 10 °C [34].

### Soil sampling for carbon analysis

**Fig. 1:**
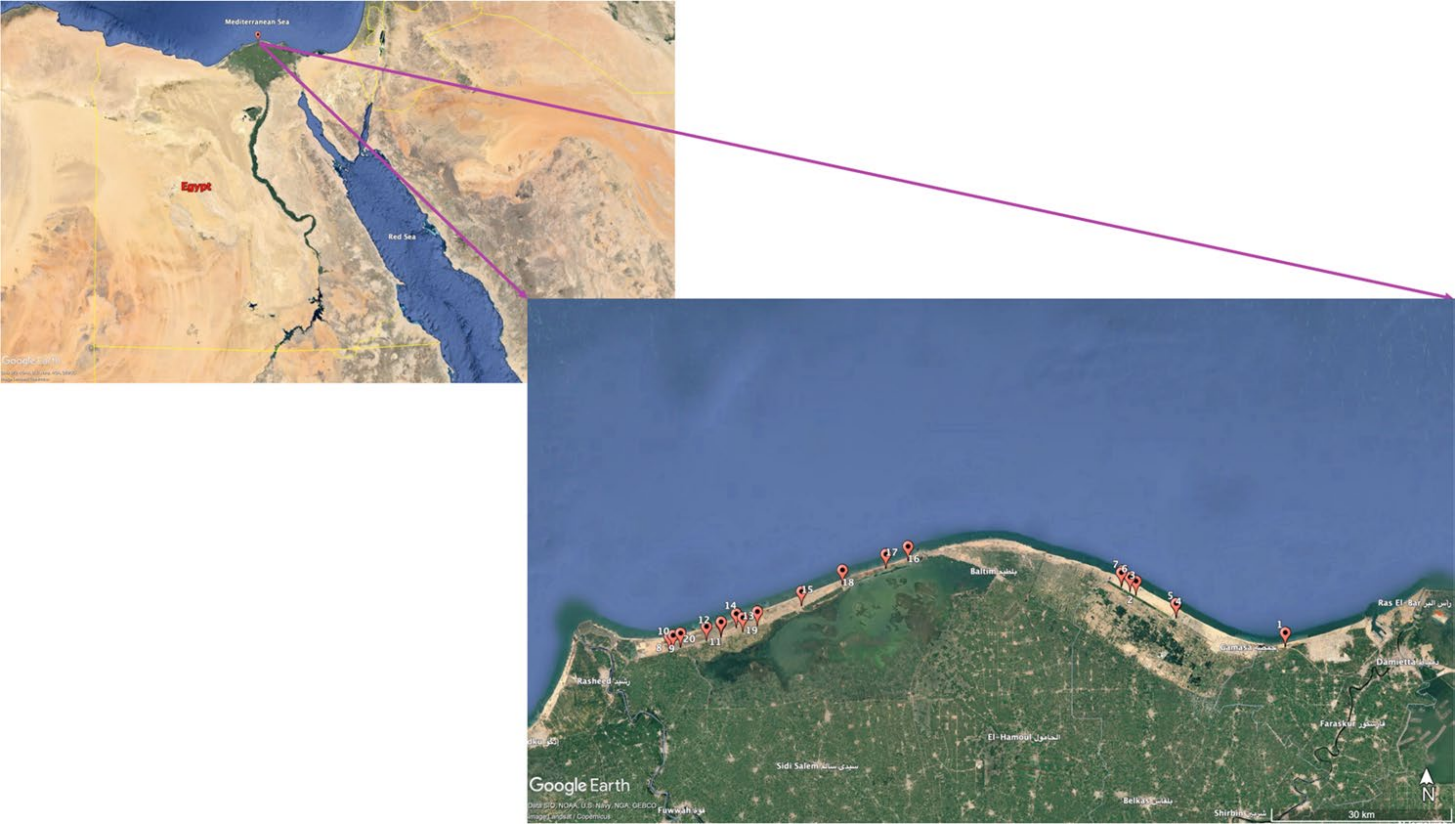
Satellite images of the study area indicate the sampling locations () of salt marshes with different plant species along the deltaic coast of Egypt (prepared using Google Earth). Exact location coordinates are provided in Table S1

This research was carried out at 20 coastal salt marsh locations along the deltaic shoreline of Egypt during January and February 2024 (Fig. 1). These specific locations were selected based on accessibility and minimal anthropogenic disturbance, distributed across roughly 120 km of the northern Delta coast. The dominant halophytic species present in the area included *Arthrocnemum macrostachyum* (Moric.) K. Koch, *Halocnemum strobilaceum* (Pall.) M. Bieb., and *Salicornia fruticosa* (L.) L. Plant specimens were taxonomically authenticated by Prof. Dr. Yassin M. Al-Sodany in accordance with the classification systems of Boulos [35] and Täckholm [36]. A voucher specimen of the plant material used in this study has been formally prepared and deposited in the Kafrelsheikh University Herbarium, indexed under the Herbarium Code KFSUH. The specimens have been assigned the following accession numbers: *Arthrocnemum macrostachyum* (Moric.) K. Koch (KFSUH-21030–21032), *Halocnemum strobilaceum* (Pall.) M. Bieb. (KFSUH-21033–21035), and *Salicornia fruticosa* (L.) L. (KFSUH-21036–21038).

At each location, soil cores were collected to capture the full range of vegetation conditions present on site. To ensure standardized sampling, monospecific stands were strictly defined as contiguous vegetation patches with a minimum area of 100 m², where the target species constituted at least 90% of the total canopy cover, as assessed visually. Some locations contained one, two, or all three dominant plant species, while others included unvegetated patches within the same site. In total, 57 soil cores were collected from unvegetated areas across 12 locations. From the defined monospecific stands, 47 cores were obtained beneath the canopy of *A. macrostachyum* across 9 locations; 51 cores from *H. strobilaceum* across 14 locations; and 45 cores from *S. fruticosa* across 11 locations (Tables S1–S2). This sampling design ensures that the dataset represents both vegetated and unvegetated conditions across the coastal salt marshes of the northern Delta. The soil cores were collected randomly using a stainless-steel hand corer measuring 100 cm in length with a 70 mm internal diameter. All soil cores were collected to a depth of 50 cm, which represented the maximum sampling depth because a thick, compact salt crust was encountered below this level, preventing further penetration of the corer. Each core was then sectioned immediately into 5 cm increments with a sharp blade, producing samples representing the depth intervals 0–5, 5–10, 10–15, 15–20, 20–25, 25–30, 30–35, 35–40, 40–45, and 45–50 cm. Each segment was transferred into a plastic container, sealed with Parafilm, and stored on ice to suppress microbial activity and reduce the risk of carbon loss before laboratory processing. In total, 200 soil cores were collected, generating 2,000 individual soil samples.

### Sample analysis

Each soil sample was oven‑dried at 105 °C for three days and then weighed to determine soil bulk density (SBD; g/cm³), following the method of Wilke [37]:

1 $$\rho_{\;{sj}}=\frac{{m}{_j}}{{v}{_j}}$$

In this formula, *ρ*_*sj*_ is the SBD of the *j*^th^ soil layer (g/cm³), *m*_*j*_ is the dry mass of the sample from that layer (g), and *v*_*j*_ represents the corresponding soil volume (cm³).

After drying, all samples were ground and sieved to ensure particle sizes were < 2 mm. Soil organic matter (SOM; %) was quantified using the loss-on-ignition procedure, where samples were combusted at 550 °C for two hours, following Jones [38]. Soil organic carbon (SOC) content was then estimated using the nonlinear conversion equation proposed by Maxwell et al. [39]:

2 $$\it \mathrm{SOC}\;\mathrm{content}\;(\%) = (0.41\times\mathrm{SOM})+(0.000683\times\mathrm{SOM}^2)$$

Volumetric SOC density (SOC_*v*_; kg C/m³) was calculated by multiplying SOC content by the measured SBD, following Bai et al. [7]:

3 $$SOC_{vj}=\rho_{sj}\times{SOC}_j$$

Here, *SOC*_*vj*_ represents the volumetric SOC density in the *j*ᵗʰ layer (kg C/m³), *ρ*_*sj*_ is the SBD (g/cm³), and *SOC*_*j*_ is the SOC content (g C/kg).

SOC stock (kg C/m²) for each soil layer was derived using the approach outlined by Bai et al. [7]:

4 $$SOC_{sj}={SOC}_{vj}\times{H}$$

where *SOC*_*sj*_ is the SOC stock for the *j*ᵗʰ layer, *SOC*_*vj*_ is the volumetric SOC density (kg C/m³), and *H* is the thickness of the layer (m).

Cumulative SOC stock (SOC_*c*_) from the surface (0–5 cm) to any specified depth was computed by summing the SOC stock across the successive layers [7]:

5 $${SOC}_{c}=\sum_{j=1}^{n}{SOC}_{sj}$$

Thus, SOC_*c*_ expresses the cumulative SOC stock (kg C/m²) down to the chosen depth.

For unvegetated marshes, 47 soil cores were assigned to calibration and 10 completely independent cores to validation. The calibration and validation sets for the vegetation types were similarly separated as follows: *A. macrostachyum* (37 calibration and 10 validation cores), *H. strobilaceum* (41 and 10 cores), and *S. fruticosa* (35 and 10 cores). To describe vertical patterns of SOC_*c*_ and SOC_*v*_, three depth-response models (allometric, exponential, and sigmoid) were fitted exclusively to the calibration datasets. Specifically, model calibration utilized the entire depth profile (0–50 cm) of the calibration cores to generate the predictive equations (presented in Tables 3 and 5, and Figs. 5 and 6). The validation process was conducted using only the separate validation dataset. Measurements from the upper seven layers (0–35 cm) of each validation core were used to predict SOC_*v*_ and SOC_*c*_ values for the bottom three layers (35–40, 40–45, and 45–50 cm). These predictions were then compared against the actual measured values, yielding 30 validation observations per vegetation condition (10 cores × 3 layers). The validation results are presented in Tables 4 and 6.

In this study, we selected the allometric, exponential, and sigmoid models because these functions are widely recognized for their effectiveness in representing vertical SOC distribution patterns in salt marsh ecosystems [22, 26]. Each model captures a distinct ecological or mathematical behavior relevant to soil carbon dynamics in salt marsh systems [7]. The allometric model reflects a nonlinear decay pattern, consistent with the rapid loss of fresh organic inputs near the soil surface and the decreasing influence of plant-derived material with depth [22]. The exponential model is well suited to representing the steep initial decline in SOC commonly observed in anoxic, waterlogged sediments, followed by gradual stabilization in deeper, more mineral-associated layers [22, 40]. The sigmoid model effectively describes SOC profiles that exhibit three phases: a rapid decline near the surface, a mid-depth transition zone, and asymptotic stabilization at greater depths, capturing the combined influences of sedimentation rates, hydrological gradients, and root-zone processes [22, 40]. These models have been successfully applied in previous SOC studies across coastal wetlands and salt marshes and have demonstrated strong performance in characterizing depth-dependent carbon dynamics. Their combined inclusion enables a comprehensive evaluation of SOC behavior and enhances the robustness of model comparisons across plant species and environmental conditions. The functions are:

6 $$\it \mathrm{y}=\mathrm{y}_0+{a}\times\mathrm{x}^b$$

7 $$\it \mathrm{y}=\mathrm{y}_0+{a}\times\mathrm{exp}^{-({b}\times\mathrm{x})}$$

8 $$\it \it \mathrm{y}=\mathrm{y}_0+\frac{a}{1+{(\mathrm{exp}^{-((\mathrm{x}-\mathrm{x}_0)/b)})}}$$

Equation (6) represents the allometric model, (7) the exponential model, and (8) the sigmoid (logistic) model.

Model performance was assessed using mean normalized average error (MNAE), mean normalized bias (MNB), and residual mean squares (RMS), based on Novotná et al. [41] and Bai et al. [7]:

9 $$MNAE=\frac{\sum\:\left(\frac{|{SOC}_{model}\:-\:{SOC}_{measured}|}{{SOC}_{measured}}\right)}{n}$$

10 $$MNB=\frac{\sum\:\left({SOC}_{model}\:-\:{{SOC}_{measured}}\right)}{\sum\:{SOC}_{measured}}$$

11 $${RMS}=\frac{\sum\:{({SOC}_{model}\:-\:{SOC}_{measured})}^{2}}{Degrees\:of\:freedom}$$

In these equations, SOC_*model*_ denotes the predicted SOC values, SOC_*measured*_ refers to field-measured SOC, and *n* is the number of samples used for validation within each vegetation category.

### Soil samples for physical and chemical analyses

At each sampling location, three soil subsamples were randomly taken from the upper 0–20 cm layer for subsequent physical and chemical assessments. The collected soils were air-dried for approximately two weeks, then finely ground and passed through a 2-mm sieve. Particle size fractions (sand, silt, and clay) were determined using the sieve-based procedure outlined by Gee and Bauder [42]. Measurements of electrical conductivity (EC) and pH were performed on a 1:2.5 soil-to-water suspension using a portable multi-parameter salinity meter. Total phosphorus and nitrogen contents were quantified spectrophotometrically using the ammonium-molybdate and indophenol blue methods, respectively, while potassium contents were analyzed using inductively coupled plasma-optical emission spectrometry (ICP-OES). All analytical steps followed the standard methods described by Allen [43].

### Plant species characteristics

At each sampling site, the density, height, and canopy width of the dominant plant species were measured within three randomly placed quadrats, each measuring 10 × 10 m. Canopy width was assessed using a measuring tape by taking three perpendicular diameter measurements through the center of the shoot, and the mean of these values was used for analysis. From each quadrat, five individuals were randomly selected (resulting in a total of 15 plants per site) to capture the natural variation in shoot growth. These specimens were then transported to the laboratory for detailed measurements. In the laboratory, shoot height and root depth were recorded, after which shoots and roots were separated, oven-dried at 60 °C, and weighed once a constant mass was reached.

### Statistical analysis

To model the vertical distribution patterns of SOC_*v*_ and SOC_*c*_, allometric, exponential, and logistic functions were developed using SigmaPlot 14.0. These functions were fitted to the aggregated calibration data, rather than individual core observations, to capture the generalized depth profile for each vegetation condition. The model fitting was performed using a nonlinear least squares optimization procedure based on the Marquardt-Levenberg algorithm. Initial parameter values required for algorithm convergence were auto-initialized by the software based on the data boundaries of the aggregated datasets. Before conducting any statistical tests, the dataset was examined for normality using the Shapiro-Wilk test and for homogeneity of variance using Levene’s test. When these assumptions were not met, non-parametric analyses were applied: the Kruskal-Wallis test for independent comparisons and the Friedman test for repeated-measures data. A two-way repeated-measures ANOVA was employed to evaluate differences in SBD, SOC content, SOC_*c*_, and SOC_*v*_ across site categories (unvegetated, *A. macrostachyum*, *H. strobilaceum*, and *S. fruticosa*) and soil depths. The relationship between SBD and SOC content was analyzed using nonlinear regression following the method described by Shari et al. [19]. One-way ANOVA was used to test for differences in soil characteristics such as pH, EC, texture fractions (sand, silt, clay), and nutrient contents (N, P, K) among the study sites. Additionally, one-way ANOVA was conducted to identify significant differences in morphological and biomass traits among the three plant species (*A. macrostachyum*, *H. strobilaceum*, and *S. fruticosa*). Where ANOVA detected significant variation, Tukey’s Honest Significant Difference (HSD) test was used for post-hoc pairwise comparisons, with significance set at *p* < 0.05. All statistical procedures were carried out using SPSS version 23.0 [44].

## Results

### Soil characteristics

**Fig. 2:**
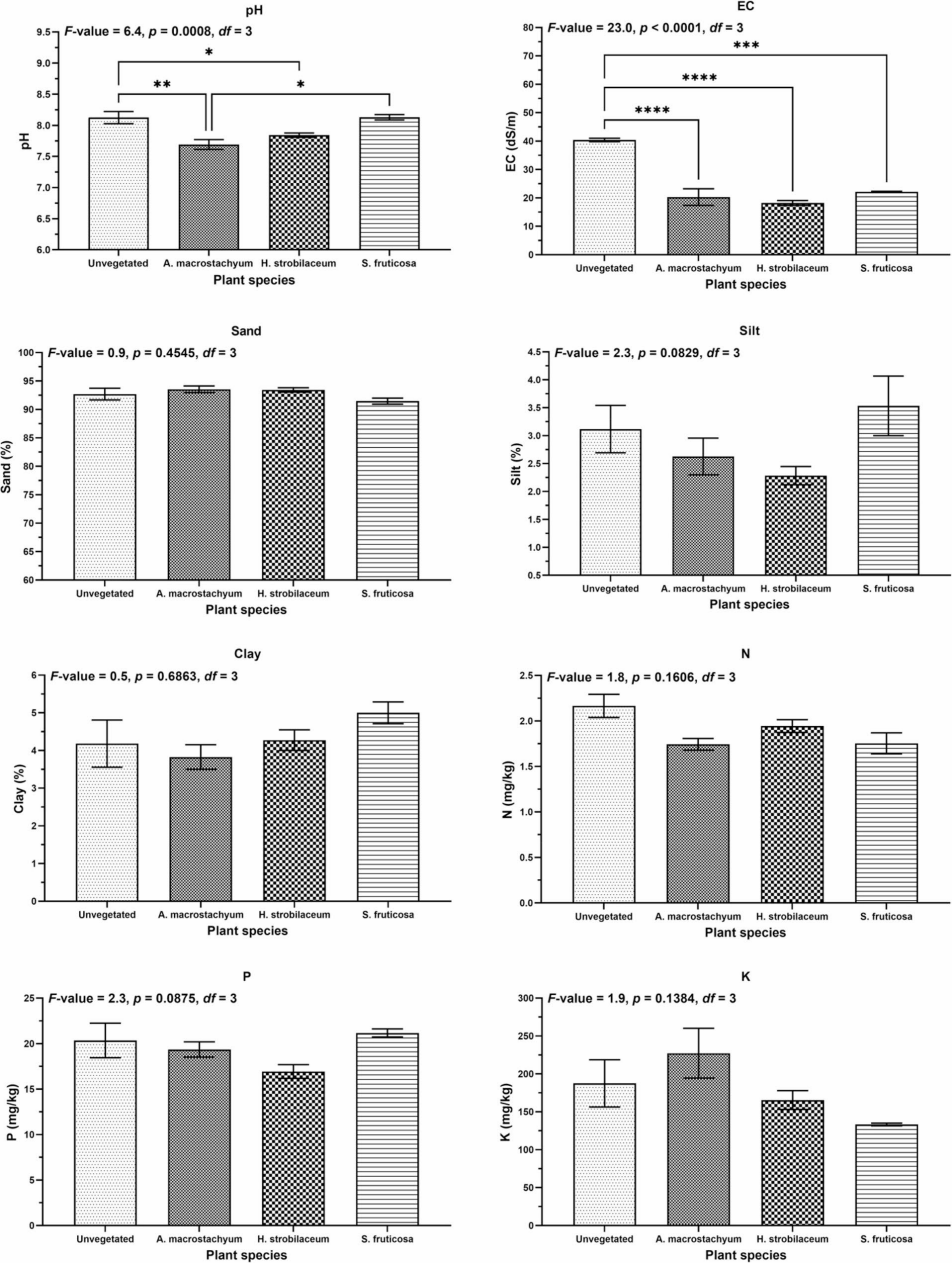
Variation in soil characteristics in salt marshes with different plant species along the deltaic coast of Egypt. Vertical bars represent the standard errors of the means. F-values are derived from one-way analysis of variance (ANOVA); df indicates degrees of freedom. Statistically significant comparisons are marked on the graphs. *: *p* < 0.05; **: *p* < 0.01; ***: *p* < 0.001; ****: *p* < 0.0001

Soil pH (*F* = 6.4, *p* < 0.001) and EC (*F* = 23.0, *p* < 0.0001) showed significant variation among the studied salt marsh sites (Fig. 2). However, sand (*F* = 0.9, *p* = 0.455), silt (*F* = 2.3, *p* = 0.083), and clay (*F* = 0.5, *p* = 0.686) contents, as well as nutrient levels (N: *F* = 1.8, *p* = 0.161; P: *F* = 2.3, *p* = 0.088; K: *F* = 1.9, *p* = 0.138), did not differ significantly across the sites (Fig. 2). Unvegetated soils exhibited the highest pH (8.13) and EC (40.4 dS/m). In contrast, sites dominated by *A. macrostachyum* had the lowest pH (7.69), while *H. strobilaceum* sites recorded the lowest EC (18.2 dS/m).

### Plant species characteristics

**Fig. 3:**
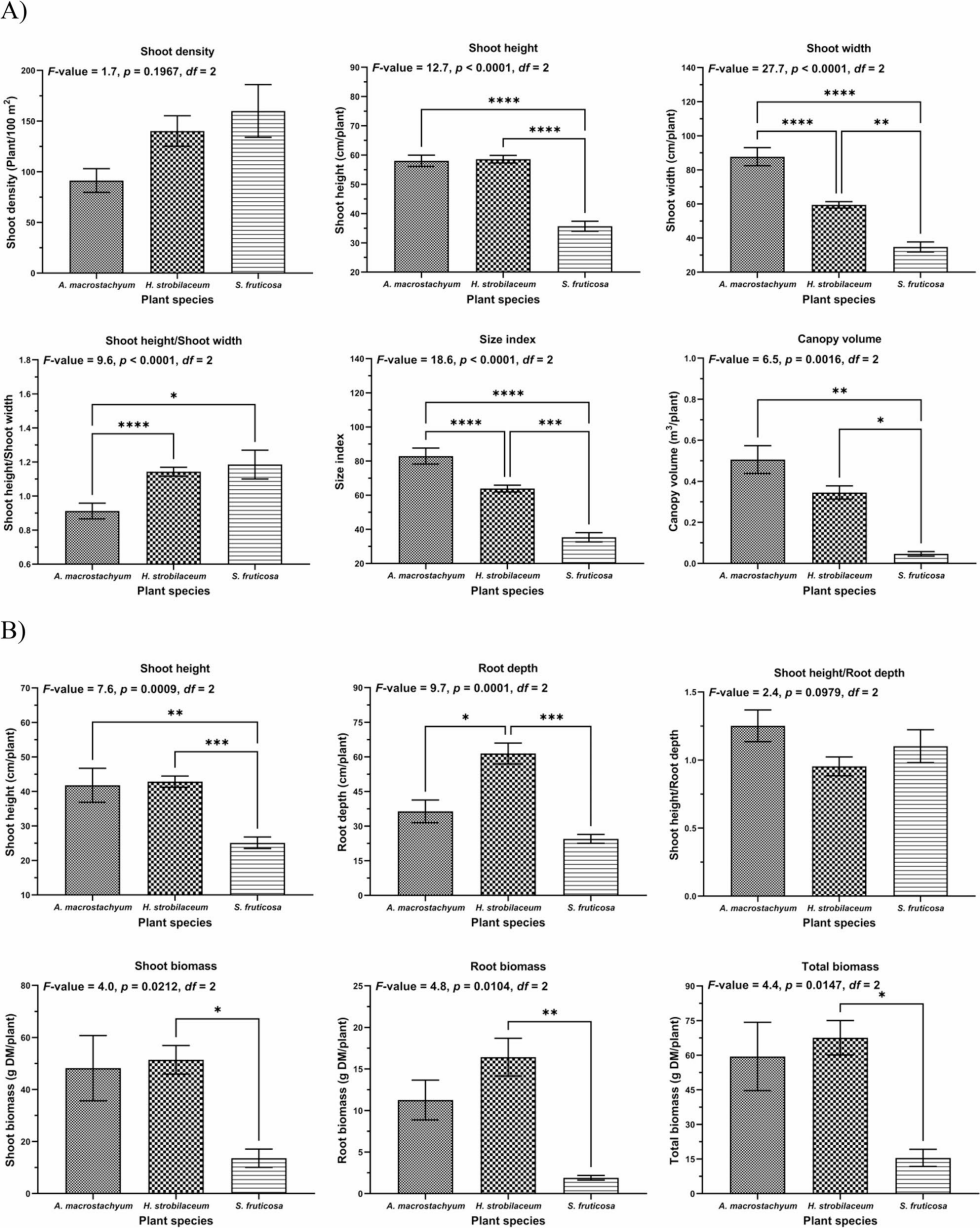
Characteristics of the three dominant plant species in salt marshes along the deltaic coast of Egypt. Vertical bars indicate the standard errors of the means. **A**: field data; **B**: lab data; F-values represent the results of the one-way analysis of variance (ANOVA); df = degrees of freedom. Statistically significant comparisons are marked on the graphs. *: *p* < 0.05; **: *p* < 0.01; ***: *p* < 0.001; ****: *p* < 0.0001

The three dominant plant species in salt marshes along Egypt’s deltaic coast exhibited significant variation in all morphological and biomass characteristics (*p* < 0.05–0.0001; Fig. 3), except for shoot density and the shoot height/root depth ratio, which were not statistically significant (*p* > 0.05; Fig. 3). *Arthrocnemum macrostachyum* had the highest shoot width (87.7 cm/plant), size index (82.9), and canopy volume (0.51 m³/plant). *Halocnemum strobilaceum* recorded the highest shoot height (58.6 and 42.8 cm/plant), root depth (61.4 cm/plant), shoot biomass (51.4 g DM/plant), root biomass (16.4 g DM/plant), and total biomass (67.6 g DM/plant). In contrast, *S. fruticosa* exhibited the lowest values across all measured traits, including shoot height (35.7 and 25.1 cm/plant), shoot width (34.8 cm/plant), size index (35.4), canopy volume (0.05 m³/plant), root depth (24.5 cm/plant), shoot biomass (13.6 g DM/plant), root biomass (1.9 g DM/plant), and total biomass (15.5 g DM/plant). Additionally, *A. macrostachyum* had the lowest shoot height/shoot width ratio (0.91), while *S. fruticosa* had the highest (1.18).

### Soil bulk density (SBD) and soil organic carbon (SOC) content

**Fig. 4:**
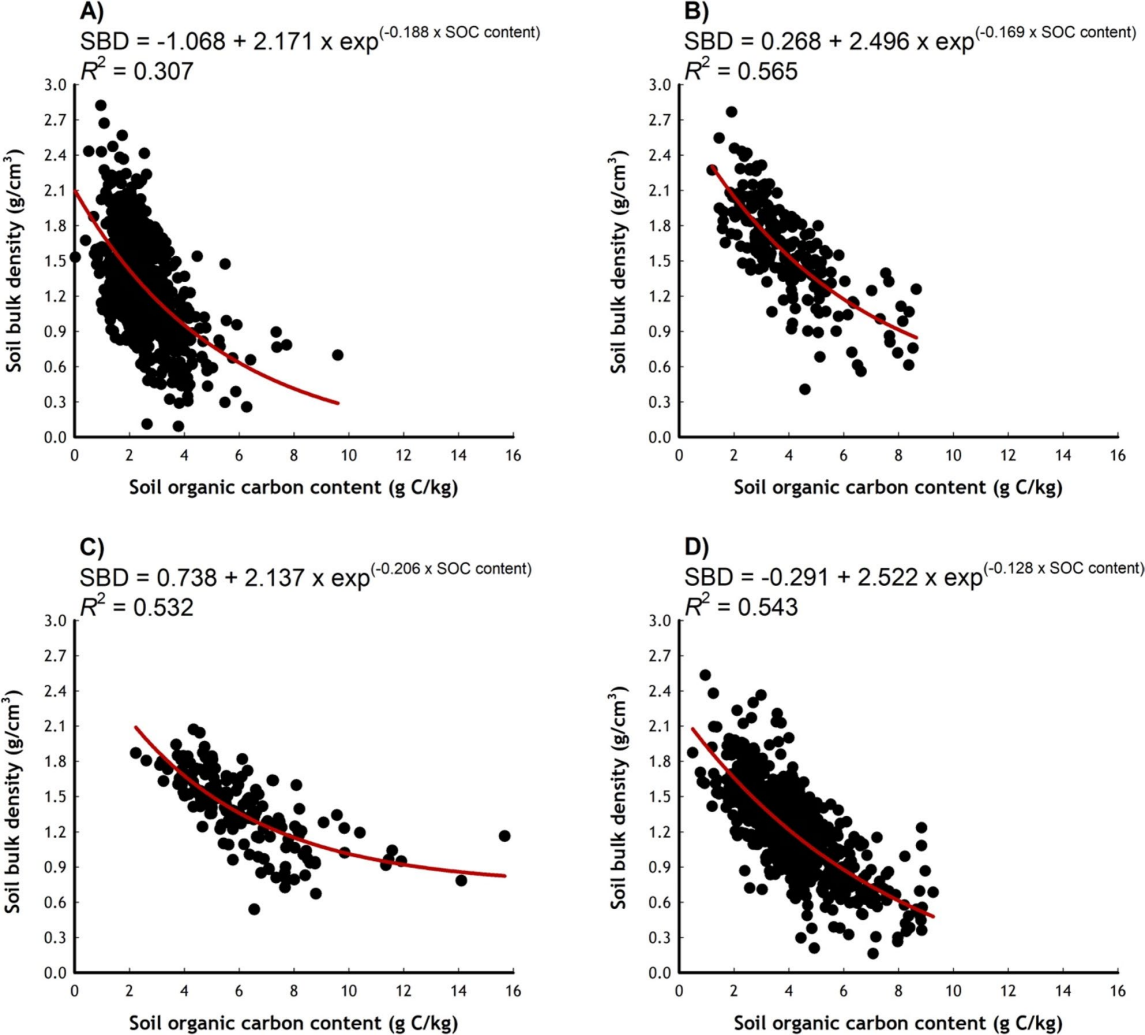
Non-linear regression between soil organic carbon (SOC) content (g C/kg) and soil bulk density (SBD; g/cm³) of soil samples in salt marshes with different plant species along the deltaic coast of Egypt. **A**) Unvegetated sites; **B**) *Arthrocnemum* *macrostachyum* sites; **C**) *Halocnemum strobilaceum* sites; and **D**) *Salicornia fruticosa* sites

The distribution of SBD at sites dominated by *A. macrostachyum* showed a significant increase from 0.91 g/cm³ at a depth of 0–5 cm to 2.14 g/cm³ at 45–50 cm (Table 1). Similarly, sites with *H. strobilaceum* exhibited an increase from 0.88 g/cm³ to 1.82 g/cm³ across the same depth range. Sites dominated by *S. fruticosa* showed an increase from 0.67 g/cm³ to 1.76 g/cm³, while unvegetated sites increased from 0.73 g/cm³ to 1.79 g/cm³ (Table 1). In contrast to SBD, SOC content decreased with increasing depth across all sampling sites. For example, in unvegetated sites, SOC content declined by 63%, from 4.11 g C/kg at the surface to 1.51 g C/kg at a depth of 45–50 cm. Similar reductions were observed in vegetated sites: a 67% decrease for *A. macrostachyum* (from 6.43 to 2.13 g C/kg), a 57% decrease for *H. strobilaceum* (from 9.49 to 4.11 g C/kg), and a 63% decrease for *S. fruticosa* (from 6.34 to 2.34 g C/kg) at the same depth (Table 2). A negative correlation between SOC content and SBD was evident at all sites, as illustrated by the nonlinear regression equations in Fig. 4: Unvegetated sites (Fig. 4A): SBD = − 1.068 + 2.171 × exp^(–0.188 × SOC content)^ (*R²* = 0.307); *A. macrostachyum* (Fig. 4B): SBD = 0.268 + 2.496 × exp^(–0.169 × SOC content)^ (*R²* = 0.565); *H. strobilaceum* (Fig. 4C): SBD = 0.738 + 2.137 × exp^(–0.206 × SOC content)^ (*R²* = 0.532); and *S. fruticosa* (Fig. 4D): SBD = − 0.291 + 2.522 × exp^(–0.128 × SOC content)^ (*R²* = 0.543). Overall, the graphs illustrate an inverse exponential relationship between SOC content and SBD across the different vegetation types.

**Table 1:** Statistical summary of soil bulk density (g/cm³) at varying depth profiles in salt marshes with different plant species along the deltaic coast of Egypt

Soil depth (cm)	Plant species															
	Unvegetated				*Arthrocnemum macrostachyum*				*Halocnemum strobilaceum*				*Salicornia fruticosa*			
	Mean(*n* = 57)	Min	Max	CV (%)	Mean(*n* = 47)	Min	Max	CV (%)	Mean(*n* = 51)	Min	Max	CV (%)	Mean(*n* = 45)	Min	Max	CV (%)
0–5	0.73	0.09	1.24	37.4	0.91	0.41	1.26	25.1	0.88	0.54	1.19	19.2	0.67	0.16	1.23	34.1
5–10	0.98	0.44	1.54	24.2	1.20	0.61	1.65	19.6	1.06	0.85	1.34	14.8	0.88	0.38	1.28	23.5
10–15	1.10	0.45	1.66	21.1	1.38	1.03	1.73	12.3	1.20	0.97	1.60	13.8	1.03	0.57	1.40	18.8
15–20	1.21	0.75	1.75	18.7	1.51	1.25	1.97	11.3	1.30	0.99	1.64	11.1	1.13	0.59	1.54	17.3
20–25	1.28	0.86	1.86	18.1	1.62	1.42	2.03	10.6	1.37	1.16	1.69	9.1	1.23	0.68	1.55	15.1
25–30	1.36	0.92	1.91	17.4	1.70	1.47	2.14	11.5	1.45	1.28	1.73	7.4	1.31	0.73	1.69	14.0
30–35	1.45	0.98	2.24	18.0	1.78	1.48	2.31	11.7	1.51	1.30	1.79	7.6	1.40	0.75	1.75	12.1
35–40	1.55	1.01	2.41	19.1	1.90	1.62	2.46	12.7	1.59	1.38	1.81	7.7	1.49	0.95	2.00	12.9
40–45	1.60	1.01	2.27	18.1	1.94	1.64	2.39	10.5	1.69	1.47	1.87	7.5	1.61	1.18	2.13	13.2
45–50	1.79	1.14	2.82	20.9	2.14	1.77	2.77	11.9	1.82	1.60	2.07	7.5	1.76	1.32	2.53	15.4
F_Species_ = 11.9***, F_Depth_ = 206.1***, F_Species × Depth_ = 1.6*																

*F*-values represent a two-way repeated measures Analysis Of Variance (ANOVA) results

*Min* Minimum, *Max:* Maximum, *CV* Coefficient of Variation

*: *p* < 0.05; ***: *p* < 0.001

**Table 2:** Statistical summary of soil organic carbon content (g C/kg) at varying depth profiles in salt marshes with different plant species along the deltaic coast of Egypt

Soil depth (cm)	Plant species															
	Unvegetated				*Arthrocnemum macrostachyum*				*Halocnemum strobilaceum*				*Salicornia fruticosa*			
	Mean(*n* = 57)	Min	Max	CV (%)	Mean(*n* = 47)	Min	Max	CV (%)	Mean(*n* = 51)	Min	Max	CV (%)	Mean(*n* = 45)	Min	Max	CV (%)
0–5	4.11	2.24	9.60	29.9	6.43	3.39	8.65	25.7	9.49	6.55	15.69	27.5	6.34	3.71	9.27	25.3
5–10	3.26	1.99	5.49	20.4	5.16	2.92	7.67	24.1	7.80	5.62	11.45	18.2	5.32	3.03	8.65	23.9
10–15	2.92	1.66	4.47	18.7	4.51	2.89	7.54	24.3	7.30	5.43	9.85	15.5	4.67	2.58	8.21	23.6
15–20	2.71	1.49	3.88	17.8	4.10	2.88	5.82	21.1	6.61	5.31	9.83	16.9	4.29	2.90	6.70	21.6
20–25	2.50	1.42	3.36	18.8	3.86	2.61	5.40	21.6	6.00	4.58	9.08	18.8	3.99	2.64	6.63	21.7
25–30	2.31	1.33	3.20	19.5	3.53	2.59	4.95	20.2	5.60	4.33	8.20	18.2	3.66	2.40	6.26	22.3
30–35	2.16	1.30	3.16	19.3	3.29	2.28	4.82	22.6	5.23	3.24	7.21	18.8	3.36	1.81	6.13	25.1
35–40	1.97	0.80	2.94	21.1	2.91	2.02	4.60	22.9	4.97	3.11	6.49	18.3	3.12	1.72	4.55	23.5
40–45	1.74	0.02	2.68	24.9	2.73	1.69	4.13	22.0	4.51	2.62	6.12	18.4	2.76	1.26	4.44	27.1
45–50	1.51	0.39	2.61	29.0	2.13	1.20	3.30	26.7	4.11	2.23	5.08	17.7	2.34	0.49	4.02	34.3
F_Species_ = 59.6***, F_Depth_ = 91.0***, F_Species × Depth_ = 2.1**																

*F*-values represent a two-way repeated measures Analysis Of Variance (ANOVA) results

*Min* Minimum, *Max* Maximum, *CV* Coefficient of Variation

**: *p* < 0.01; ***: *p* < 0.001

### Fitting models for SOC_v_ and SOC_c_

**Fig. 5:**
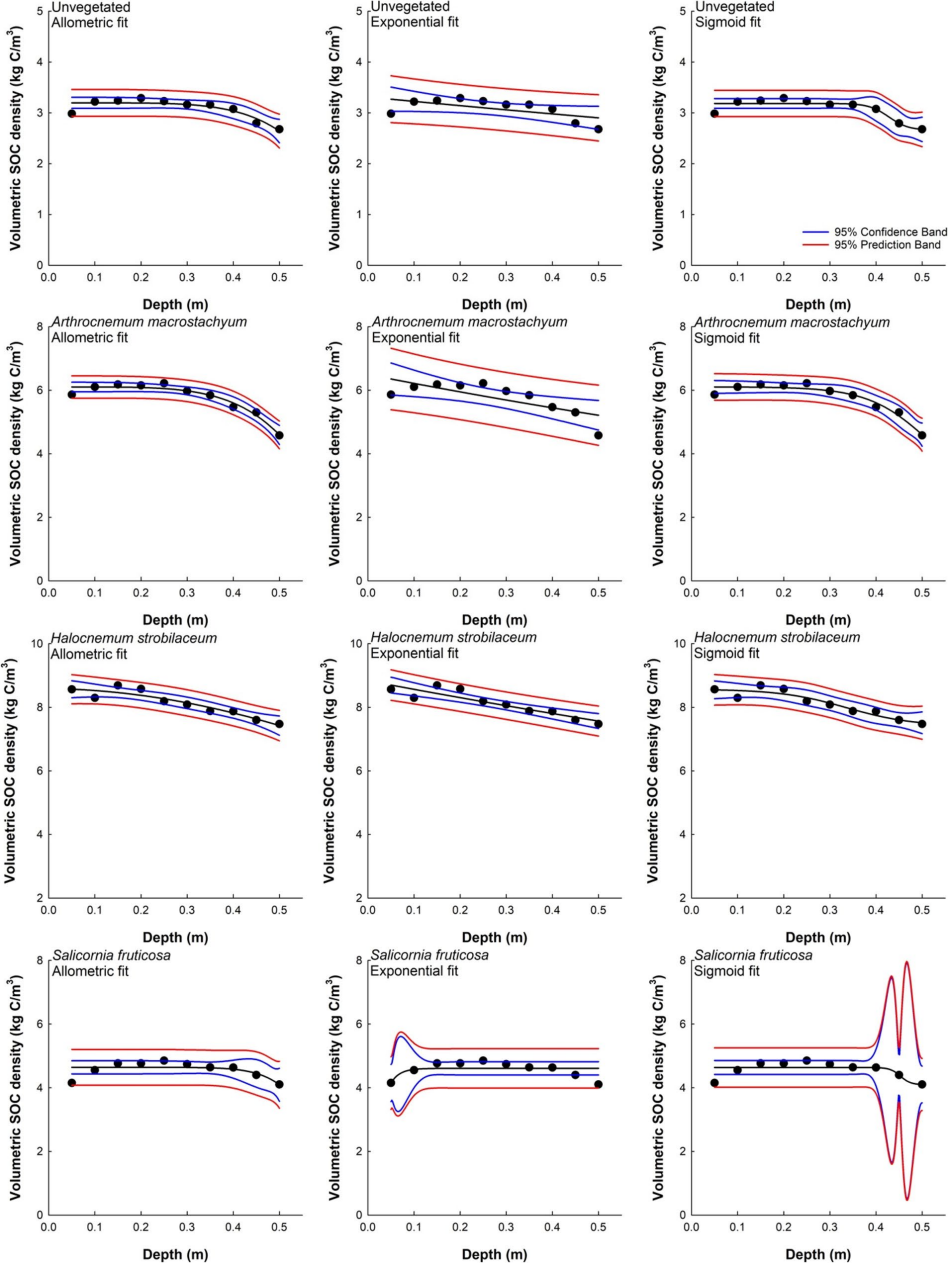
Curve-fitting models for volumetric soil organic carbon density (SOCv; kg C/m³) as a function of soil profile (m) in salt marshes with different plant species along the deltaic coast of Egypt. The black line represents the regression model, while the blue and red lines indicate the 95% confidence and prediction bands, respectively

Two-way repeated-measures ANOVA revealed significant differences in SOC_*v*_ among species (*F* = 41.4, *p* < 0.001) and with depth (*F* = 3.1, *p* < 0.01), but the species × depth interaction was not significant (*F* = 0.7, *p* > 0.05). Sites dominated by *H. strobilaceum* recorded the highest mean SOC_*v*_ values (8.12 ± 0.14 kg C/m³), while *A. macrostachyum* (5.79 ± 0.10 kg C/m³) and *S. fruticosa* (4.56 ± 0.05 kg C/m³) exhibited intermediate values. Unvegetated sites had the lowest SOC_*v*_ (3.09 ± 0.03 kg C/m³) (Fig. 5), consistent with the proposed hypothesis. Overall, the figure visually confirms that SOC_*v*_ generally decreases with increasing soil depth across all vegetation types. It highlights the influence of both soil depth and plant species on SOC accumulation, emphasizing the ecological importance of vegetation in shaping soil properties in salt marsh environments.

Table 3 presents the parameters and goodness-of-fit metrics for three mathematical formulas (allometric, exponential, and sigmoid) used to model SOC_*v*_ across different salt marsh vegetation states, while Fig. 5 presents the 95% confidence and prediction bands. The calibration success varies significantly depending on the dominant plant species and the chosen formula: Across almost all vegetation states, the exponential models consistently exhibit the weakest calibration, yielding the lowest *R²* and Adjusted *R²* values, and generally higher RMS. The allometric and sigmoid models demonstrate vastly superior predictive potential during this generation phase. *Arthrocnemum macrostachyum*: The allometric model shows an exceptionally strong goodness of fit for this species, achieving the highest *R²* (0.9482) and the most negative Akaike Information Criterion Corrected (AICc) score (–27.8077) among all models tested for this plant. Unvegetated and *H. strobilaceum* soils: For these areas, the sigmoid model provides the highest *R²* values (0.8463 and 0.9020, respectively). However, the allometric model also performs very competitively, especially regarding the AICc scores. *Salicornia fruticosa*: The predictive models for this species display comparatively weak calibration across all three formulas, with *R²* values remaining below 0.46. The sigmoid model performed marginally better (*R²* = 0.4561) than the allometric model (*R²* = 0.4460).

**Table 3:** Model parameters for volumetric soil organic carbon density (SOC_*v*_; kg C/m³) using three formulas for salt marshes with different plant species along the deltaic coast of Egypt. The goodness of fit for the generated models is indicated

Plant species	Allometric	Exponential	Sigmoid
Unvegetated	*y* = 3.1978–26.1070 × ($\:{x}^{5.5537}$)	*y* = 3.3128 × ($\:{exp}^{-0.2644\:\times\:\:x}$)	*y* = 2.6586 + $\:\frac{0.5269}{1\:+\:\left({exp}^{-\left(\right(x-0.4276)/-0.0214)}\right)}$
	*R*^*2*^ = 0.8085	*R*^*2*^ = 0.3780	*R*^*2*^ = 0.8463
	*Adj. R*^*2*^ = 0.7538	*Adj. R*^*2*^ = 0.3003	*Adj. R*^*2*^ = 0.7693
	*RMS =* 0.0103	*RMS =* 0.0293	*RMS =* 0.0097
	*AICc =* − 33.2979	*AICc =* − 27.5185	*AICc =* − 26.4964
*Arthrocnemum macrostachyum*	*y* = 6.1032–45.9709 × ($\:{x}^{4.9291}$)	*y* = 6.4966 × ($\:{exp}^{-0.4400\:\times\:\:x}$)	*y* = 2.7100 + $\frac{3.3981}{1\:+\:\left({exp}^{-\left(\right(x-0.5151)/-0.0656)}\right)}$
	*R*^*2*^ = 0.9482	*R*^*2*^ = 0.5756	*R*^*2*^ = 0.9442
	*Adj. R*^*2*^ = 0.9334	*Adj. R*^*2*^ = 0.5225	*Adj. R*^*2*^ = 0.9163
	*RMS =* 0.0179	*RMS =* 0.1282	*RMS =* 0.0225
	*AICc =* − 27.8077	*AICc =* − 12.7767	*AICc =* − 18.0623
*Halocnemum strobilaceum*	*y* = 8.5862–4.2672 × (${x}^{1.8783}$)	*y* = 8.8377 × (${exp}^{-0.3101\:\times\:\:x}$)	*y* = 7.4084 + $\frac{1.1642}{1\:+\:\left({exp}^{-\left(\right(x-0.3359)/-0.0717)}\right)}$
	*R*^*2*^ = 0.8907	*R*^*2*^ = 0.8388	*R*^*2*^ = 0.9020
	*Adj. R*^*2*^ = 0.8595	*Adj. R*^*2*^ = 0.8186	*Adj. R*^*2*^ = 0.8531
	*RMS =* 0.0246	*RMS =* 0.0317	*RMS =* 0.0257
	*AICc =* − 26.7314	*AICc =* − 16.7127	*AICc =* − 24.6188
*Salicornia fruticosa*	*y* = 4.6403–562.3604 × (${x}^{10.0008}$)	*y* = 4.6083 − 6.3741 × ($\:{exp}^{-52.5022\:\times\:\:x}$)	*y* = 4.6360 + $\frac{0.5420}{1\:+\:\left({exp}^{-\left(\right(x-0.4529)/0.0112)}\right)}$
	*R*^*2*^ = 0.4460	*R*^*2*^ = 0.3078	*R*^*2*^ = 0.4561
	*Adj. R*^*2*^ = 0.2877	*Adj. R*^*2*^ = 0.1100	*Adj. R*^*2*^ = 0.1841
	*RMS =* 0.0487	*RMS =* 0.0609	*RMS =* 0.0558
	*AICc =* − 17.7801	*AICc =* − 15.5529	*AICc =* − 8.9643

*R*^2^ Coefficient of Determination, *Adj. R*^2^ Adjusted R^2^, *RMS* Residual Mean Square, *AICc* Akaike Information Criterion Corrected

Table 4 and Figure S1 assesses the robustness of the generated models by comparing predicted versus observed values. The critical metric here is the Student’s t-test *p*-value; a model is only considered fundamentally valid if there is no statistically significant difference between the predicted and observed datasets (*p* > 0.05). All exponential models struggle during validation. For both unvegetated soils (*p* = 0.008) and *S. fruticosa* (*p* = 0.000), the exponential models are statistically invalid. Furthermore, the allometric model for *S. fruticosa* is also invalid (*p* = 0.009). Among the statistically valid models, the allometric formula proves highly precise for *A. macrostachyum*, recording the lowest Mean Normalized Average Error (MNAE = 0.094) and Residual Mean Square (RMS = 0.246). Conversely, the sigmoid formula exhibits the lowest error rates for unvegetated soils (MNAE = 0.249), *H. strobilaceum* (MNAE = 0.178), and *S. fruticosa* (MNAE = 0.335).

**Table 4:** Assessment of validation indices for volumetric soil organic carbon density (SOC_*v*_; kg C/m³) using three formulas for salt marshes with different plant species along the deltaic coast of Egypt

Plant species	Formula	MNAE	MNB	RMS	Student’s *t*-test	
					*t*-value	*p*
Unvegetated (*n* = 30)	Allometric	0.267	0.119	1.506	1.784	0.079
	Exponential	0.482	0.193	8.295	2.751	0.008
	Sigmoid	0.249	0.110	0.564	1.719	0.091
*Arthrocnemum macrostachyum* (*n* = 30)	Allometric	0.094	0.066	0.246	1.485	0.143
	Exponential	0.298	0.164	2.641	1.983	0.056
	Sigmoid	0.135	-0.066	0.285	1.541	0.129
*Halocnemum strobilaceum* (*n* = 30)	Allometric	0.190	0.104	0.542	1.710	0.093
	Exponential	0.251	0.114	0.815	1.778	0.081
	Sigmoid	0.178	0.094	0.332	1.670	0.100
*Salicornia fruticosa* (*n* = 30)	Allometric	0.429	0.190	4.450	2.713	0.009
	Exponential	0.548	0.369	35.725	4.011	0.000
	Sigmoid	0.335	0.165	2.810	2.018	0.052

*MNAE* Mean Normalized Average Error, *MNB* Mean Normalized Bias, *RMS* Residual Mean Square

**Fig. 6:**
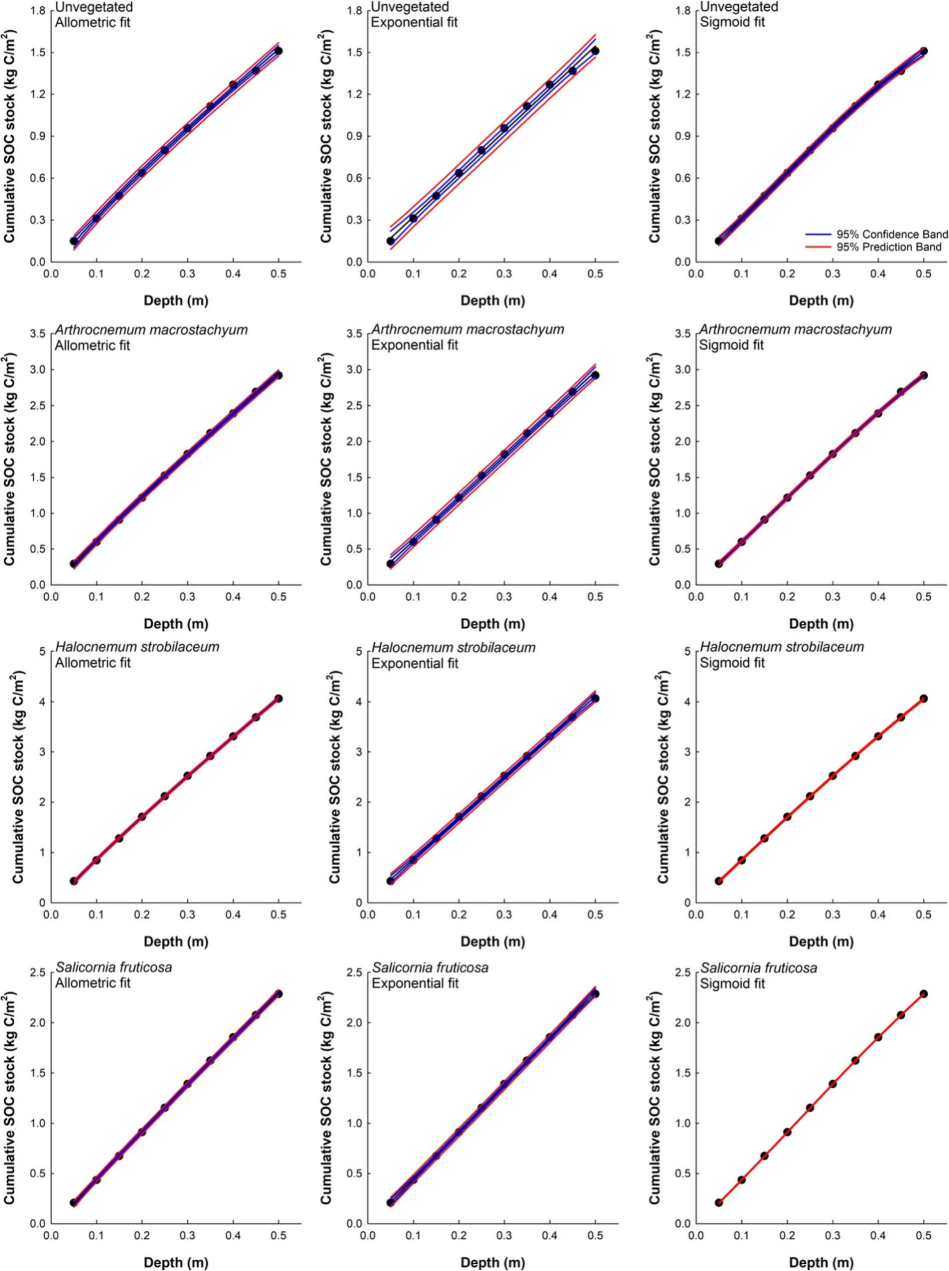
Curve-fitting models for cumulative soil organic carbon stock (SOCc; kg C/m²) as a function of soil profile (m) in salt marshes with different plant species along the deltaic coast of Egypt. The black line represents the regression model, while the blue and red lines indicate the 95% confidence and prediction bands, respectively

Two-way repeated-measures ANOVA revealed significant differences in SOC_*c*_ among species (*F* = 30.0, *p* < 0.001), with depth (*F* = 1576.7, *p* < 0.001), and for their interaction (*F* = 34.3, *p* < 0.001). Sites dominated by *H. strobilaceum* recorded the highest mean SOC_*c*_ values (4.06 ± 0.14 kg C/m²), while *A. macrostachyum* (2.92 ± 0.11 kg C/m²) and *S. fruticosa* (2.28 ± 0.05 kg C/m²) exhibited intermediate values. Unvegetated sites had the lowest SOC_*c*_ (1.51 ± 0.04 kg C/m²) (Fig. 6), further supporting the proposed hypothesis.

Table 5 presents the parameters and goodness-of-fit indicators for three formulas modeling SOC_*c*_, while Fig. 6 presents the 95% confidence and prediction bands. In contrast to typical volumetric density models, all three formulas (allometric, exponential, and sigmoid) demonstrate an exceptionally high degree of calibration fit for SOC_*c*_, with *R²* values exceeding 0.99 across all plant species and unvegetated states. Because the *R²* values are uniformly near-perfect, the AICc serves as the primary differentiator for model quality. The sigmoid models consistently yield the lowest (most negative) AICc values across all categories, indicating superior model parsimony, particularly for *S. fruticosa* (AICc = − 109.1976), *H. strobilaceum* (AICc = − 82.4686), *A. macrostachyum* (AICc = − 73.1446), and unvegetated sites (AICc = − 72.6280).

**Table 5:** Model parameters for cumulative soil organic carbon stock (SOC_*c*_; kg C/m²) using three formulas for salt marshes with different plant species along the deltaic coast of Egypt. The goodness of fit for the generated models is indicated

Plant species	Allometric	Exponential	Sigmoid
Unvegetated	*y* = 2.9195 × (${x}^{0.8615}$) – 0.0842	*y* = 116.8325 × ($\:{exp}^{0.0260\:\times\:\:x}$) – 116.8128	*y* = $\frac{3.3116}{1\:+\:\left({exp}^{-\left(\right(x-0.1798)/0.2505)}\right)}$ – 1.0860
	*R*^*2*^ = 0.9990	*R*^*2*^ = 0.9974	*R*^*2*^ = 0.9997
	*Adj. R*^*2*^ = 0.9988	*Adj. R*^*2*^ = 0.9966	*Adj. R*^*2*^ = 0.9996
	*RMS =* 0.0003	*RMS =* 0.0007	*RMS =* 9.6 × 10^–5^
	*AICc =* − 69.8810	*AICc =* − 59.9015	*AICc =* − 72.6280
*Arthrocnemum macrostachyum*	*y* = 5.7148 × (${x}^{0.9097}$) – 0.0959	*y* = 169.4702 × (${exp}^{0.0345\:\times\:\:x}$) – 169.4391	*y* = $\frac{8.5841}{1\:+\:\left({exp}^{-\left(\right(x-0.1650)/0.3466)}\right)}$ – 3.2930
	*R*^*2*^ = 0.9997	*R*^*2*^ = 0.9990	*R*^*2*^ = 0.9999
	*Adj. R*^*2*^ = 0.9996	*Adj. R*^*2*^ = 0.9987	*Adj. R*^*2*^ = 0.9999
	*RMS =* 0.0003	*RMS =* 0.0011	*RMS =* 9.1 × 10^–5^
	*AICc =* − 68.6118	*AICc =* − 56.0888	*AICc =* − 73.1446
*Halocnemum strobilaceum*	*y* = 7.8633 × ($\:{x}^{0.9187}$) – 0.0878	*y* = 219.2322 × ($\:{exp}^{0.0366\:\times\:\:x}$) – 219.1639	*y* = $\:\frac{26.002}{1\:+\:\left({exp}^{-\left(\right(x+0.1444)/0.7383)}\right)}$ – 14.2804
	*R*^*2*^ = 0.9999	*R*^*2*^ = 0.9993	*R*^*2*^ = 0.9998
	*Adj. R*^*2*^ = 0.9999	*Adj. R*^*2*^ = 0.9991	*Adj. R*^*2*^ = 1.0000
	*RMS =* 0.0001	*RMS =* 0.0013	*RMS =* 3.6 × 10^–5^
	*AICc =* − 77.6448	*AICc =* − 54.1563	*AICc =* − 82.4686
*Salicornia fruticosa*	*y* = 4.5852 × (${x}^{0.9571}$) – 0.0638	*y* = 112.4283 × ($\:{exp}^{0.0410\:\times\:\:x}$) – 112.4438	*y* = $\frac{6.7037}{1\:+\:\left({exp}^{-\left(\right(x-0.2187)/0.3488)}\right)}$ – 2.3505
	*R*^*2*^ = 0.9998	*R*^*2*^ = 0.9996	*R*^*2*^ = 0.9999
	*Adj. R*^*2*^ = 0.9998	*Adj. R*^*2*^ = 0.9995	*Adj. R*^*2*^ = 1.0000
	*RMS =* 0.0001	*RMS =* 0.0002	*RMS =* 2.5 × 10^–6^
	*AICc =* − 79.5073	*AICc =* − 70.8396	*AICc =* − 109.1976

R^2^: Coefficient of Determination; Adj. R^2^: Adjusted R^2^; RMS: Residual Mean Square; AICc: Akaike Information Criterion Corrected

Table 6 and Figure S2 confirms the predictive robustness of the generated equations. Most notably, all models across all vegetation states are fundamentally statistically valid, as every Student’s t-test *p*-value is significantly greater than 0.05. The sigmoid formula consistently produces the most precise predictions, achieving the lowest MNAE and RMS for unvegetated soils (MNAE = 0.065, RMS = 0.018), *A. macrostachyum* (MNAE = 0.020, RMS = 0.002), and *S. fruticosa* (MNAE = 0.044, RMS = 0.005). Conversely, for *H. strobilaceum*, the allometric model yields the absolute lowest error rates during testing (MNAE = 0.034, RMS = 0.003).

**Table 6:** Assessment of validation indices for cumulative soil organic carbon stock (SOC_*c*_; kg C/m²) using three formulas for salt marshes with different plant species along the deltaic coast of Egypt

Plant species	Formula	MNAE	MNB	RMS	Student’s *t*-test	
					*t*-value	*p*
Unvegetated (*n* = 30)	Allometric	0.091	-0.064	0.123	0.919	0.362
	Exponential	0.092	-0.065	0.231	1.475	0.146
	Sigmoid	0.065	0.045	0.018	0.763	0.449
*Arthrocnemum macrostachyum* (*n* = 30)	Allometric	0.061	0.042	0.011	0.747	0.458
	Exponential	0.071	0.062	0.049	0.903	0.370
	Sigmoid	0.020	-0.006	0.002	0.119	0.905
*Halocnemum strobilaceum* (*n* = 30)	Allometric	0.034	0.006	0.003	0.157	0.876
	Exponential	0.066	0.059	0.034	0.860	0.393
	Sigmoid	0.045	0.029	0.010	0.386	0.701
*Salicornia fruticosa* (*n* = 30)	Allometric	0.046	0.032	0.010	0.702	0.486
	Exponential	0.065	0.046	0.019	0.787	0.435
	Sigmoid	0.044	-0.024	0.005	0.329	0.743

*MNAE* Mean Normalized Average Error, *MNB* Mean Normalized Bias, *RMS* Residual Mean Square

## Discussion

Soil bulk density (SBD) is widely recognized as a key indicator of soil physical condition because it directly influences SOC storage, soil aeration, and moisture availability [45–46]. It also reflects mechanical resistance to root penetration and affects the spatial distribution of organic matter within soil profiles [47–48]. In this study, SBD increased consistently with depth across all vegetation types (*A. macrostachyum*, *H. strobilaceum*, *S. fruticosa*) as well as unvegetated sites. Comparing these depth profiles with other coastal salt marsh ecosystems globally reveals a universal structural pattern that transcends local climatic or geographic variations [7, 13–15, 22, 26]. The consistency of this trend across disparate systems provides a coherent interpretation: depth-related increases in SBD are fundamentally driven by shared pedological and eco-geomorphic processes rather than site-specific conditions. Specifically, as noted in comparable systems, SBD is primarily shaped by the coupled effects of progressive organic matter decline and the physical compaction associated with overlying sediment deposition [48–49]. In our study system, these universal mechanisms operate in tandem: surface layers maintain lower SBD due to continuous organic inputs and active root networks, while deeper layers become increasingly compacted and mineral-dense. Contextualizing our findings within this broader global framework underscores that the vertical stratification of SBD (and its subsequent control on SOC) is a predictable, fundamental feature of coastal wetland pedogenesis.

In our study, unvegetated sites exhibited the lowest average SOC content compared to areas dominated by *A. macrostachyum*, *H. strobilaceum*, and *S. fruticosa*. This stark contrast highlights the foundational role of autogenic organic inputs in functioning salt marsh ecosystems. Rather than viewing SOC dynamics as a collection of isolated variables, comparing our vegetated and unvegetated sites through the lens of established global models [7, 50–51] provides a coherent mechanistic interpretation. The presence of these specific halophyte communities actively engineers the soil carbon pool; they drive SOC accumulation not only through direct above-ground litterfall (such as leaves and stems) but also through integrated below-ground processes, including root architecture and biomass allocation. Consequently, the spatial and temporal variations in SOC content observed across our sites are not random, but are directly governed by the structural composition of the local plant communities interacting with baseline geomorphological conditions [52]. Ultimately, it is this continuous, dynamic balance between robust organic matter inputs from the vegetated canopy and its subsequent microbial decomposition that shapes the distinct SOC content distribution patterns in these coastal habitats [53].

In the present study, SOC content showed a consistent decline with increasing depth throughout the 0–50 cm soil profile across all sampling sites, regardless of whether the areas were unvegetated or dominated by *A. macrostachyum*, *H. strobilaceum*, or *S. fruticosa*. Rather than being a localized phenomenon, this downward trend reflects a globally consistent feature of coastal salt marsh ecosystems, where fundamental biological processes drive carbon distribution across diverse climatic and geographic conditions [13–15, 19, 53–54]. Across these various global systems, elevated SOC content levels near the surface are uniformly attributed to the continuous accumulation of plant litter. As depth increases, this carbon content typically diminishes due to the progressive breakdown of labile organic compounds and ongoing decomposition processes [11]. Comparing our system to these global studies underscores that surface-driven carbon input is the primary, overarching control on vertical SOC profiles. However, localized biogeochemical and hydrological factors can sometimes alter this standard distribution. For instance, Bai et al. [7] reported a notable exception with a SOC content peak at the 40–60 cm depth, demonstrating that site-specific mechanisms (such as the downward movement of SOC content through leaching and deep microbial activity) can drive complex vertical redistributions that deviate from the standard linear decline.

In this study, the relationship between SBD and SOC content was modeled using an exponential function, capturing the observed empirical trend of increasing SBD and concurrently decreasing SOC with depth. A strong negative correlation between SBD and SOC content was identified, demonstrating that SBD dictates essential soil physical constraints (such as aeration, structural integrity, and permeability) which directly regulate SOC accumulation [55]. Comparing our exponential model outcomes with a wide array of global studies reveals that this inverse relationship is a defining, universal feature of salt marsh ecosystems rather than a site-specific anomaly [6, 9, 11–14, 17]. Integrating these disparate studies provides a coherent mechanistic interpretation: the physical soil matrix and its carbon storage capacity are mutually and inextricably linked across diverse climatic zones. As organic matter inputs decline and mineral compaction increases with depth, the elevated SBD actively restricts the pore space and aeration required for robust carbon sequestration. Consequently, the exponential function utilized here does not merely describe local data, but mathematically represents a fundamental eco-physical feedback loop that governs carbon dynamics in coastal wetlands worldwide.

While global SOC_*c*_ in coastal ecosystems average approximately 268 Mg C/ha [56], this global mean largely reflects highly productive temperate and tropical wetlands. In contrast, the values recorded in our study (15.1 Mg C/ha in unvegetated sites, and 22.8 to 40.6 Mg C/ha in vegetated sites: *S. fruticosa*, *A. macrostachyum*, and *H. strobilaceum*) are markedly lower. Rather than being a site-specific anomaly, comparing our findings with similar systems reveals that this divergence is a coherent feature of arid and semi-arid salt marsh ecosystems [16, 19, 53, 57]. While overarching factors like geomorphology, hydrology, and tidal exchange universally govern carbon storage [18, 58], arid coastal systems are uniquely bottlenecked by extreme environmental stressors. In our study area, the combination of high salinity, elevated temperatures, negligible freshwater input, limited nutrient availability, and compacted substrates fundamentally restricts both primary productivity (reducing organic inputs) and vegetation diversity [18]. Consequently, these harsh conditions limit the functional capacity of the ecosystem to sequester carbon at rates comparable to global averages. Finally, it is important to contextualize this comparison methodologically: the global estimate integrates the top meter of soil, whereas our SOC_*c*_ estimations were constrained to a refusal depth of 50 cm, further contributing to the observed variance.

In this study, soils associated with *H. strobilaceum* exhibited the highest average SOC_*v*_ and SOC_*c*_ among the vegetated sites, followed by *A. macrostachyum*, with *S. fruticosa* recording the lowest. This clear interspecific variation can be mechanistically interpreted by comparing our species-specific morphological traits with functional carbon dynamic models established in other salt marsh ecosystems [7, 51, 59]. Our data shows that *H. strobilaceum* possessed the highest values for shoot height, root depth, and total biomass. When integrated with findings from broader coastal systems, these specific structural traits provide a dual advantage for carbon accumulation. First, robust above-ground architecture (such as the superior shoot height of *H. strobilaceum* and the expansive canopy volume of *A. macrostachyum*) acts as a physical barrier that efficiently traps suspended sediments and incorporates allochthonous organic matter [60–61]. Second, and arguably more critical, the pronounced below-ground development (root depth and biomass) of *H. strobilaceum* actively engineers the soil profile. Extensive root networks do not merely provide passive organic inputs; as demonstrated across various coastal environments, they dictate the spatial distribution of SOC_*v*_ and SOC_*c*_ by promoting soil aggregation and stimulating microbial activity deep within the sediment matrix [3, 62–63]. Therefore, the lowest SOC_*v*_ and SOC_*c*_ values observed under *S. fruticosa* directly correspond to its consistently lower morphological measurements, reinforcing the conclusion that carbon sequestration capacity in these habitats is intricately tied to the specific eco-physical footprint of the dominating halophyte species.

Selecting an appropriate mathematical model is crucial for accurately representing the complex eco-physical processes governing vertical SOC distribution. In many global salt marsh ecosystems, vertical SOC profiles are inherently nonlinear, driven by predictable depth-dependent shifts in decomposition dynamics, root biomass attenuation, and sedimentation history [7, 26]. While linear models often oversimplify these vertical gradients, the application of nonlinear models (specifically the allometric, exponential, and sigmoid functions utilized in this study) provides a robust mechanistic framework. By comparing our modeling approach with broader soil science literature [22–23], it becomes clear that these specific functions do more than simply fit data points; they mathematically capture the functional reality of carbon dynamics in coastal wetlands. For instance, these nonlinear decays effectively reflect the rapid surface-level microbial decomposition and the dense topsoil root architecture typical of these habitats. Integrating these mathematical models with our empirical data provides a coherent interpretation: the curvilinear nature of SOC depth profiles is not a site-specific artifact, but a consistent, mathematically predictable outcome of the coupled biological and geomorphological processes defining these ecosystems.

Accurate estimation and prediction of SOC_*v*_ across soil profiles are becoming increasingly vital for the successful implementation of greenhouse gas mitigation initiatives and carbon trading systems [22, 64]. To achieve this, researchers globally have employed various mathematical functions (such as exponential, allometric, and sigmoid models) to characterize vertical SOC_*v*_ profiles across diverse environments, ranging from terrestrial forests and agricultural fields to coastal salt marsh ecosystems [7, 26, 50, 65]. However, rather than viewing these models merely as statistical tools fitted to specific geographic regions, comparing their global application provides a coherent mechanistic interpretation of soil carbon dynamics. In the present study, the sigmoid model provided the most robust fit for unvegetated sites, *H. strobilaceum*, and *S. fruticosa*, whereas the allometric model was most effective for *A. macrostachyum*. This divergence in model suitability is not arbitrary; it mathematically reflects underlying eco-physical interactions. The superior fit of the allometric model in *A. macrostachyum* sites captures a power-law depth relationship, which is likely driven by specific species traits such as root architecture and rapid organic matter turnover [22]. Conversely, the effectiveness of the sigmoid model in the other sites accurately mirrors the gradual, transitional decay of SOC_*v*_ – capturing the shift from biologically active surface layers to highly stable, mineral-bound subsoil layers [66]. Furthermore, the variation in optimal predictive models observed between our study and previous literature highlights that SOC_*v*_ distribution is fundamentally shaped by localized environmental stressors (e.g., salinity, compaction, nutrient availability) and distinct halophyte community structures, rather than broad geographic location alone [7, 67]. Finally, these comparisons underscore the critical role of methodological standardization; variations in sampling intensity and coring depths inherently influence model precision and generalizability. Therefore, accurately predicting SOC_*v*_ requires integrating these biological mechanisms, environmental constraints, and methodological frameworks into a unified interpretation, rather than simply comparing statistical outputs across disparate regions.

Among the studied species, *S. fruticosa* exhibited the lowest model performance for SOC_*v*_, with the best-fitting model explaining only 45.6% of the variance. This relatively weak depth-predictive relationship indicates that vertical SOC_*v*_ distribution in these specific stands cannot be adequately captured by depth alone. When contextualizing this finding within broader salt marsh ecosystems, it becomes evident that modeling carbon dynamics in highly heterogeneous or transitional ecotonal zones frequently yields lower predictive power. Although our sample size (45 cores) is methodologically robust, *S. fruticosa* typically occupies micro-habitats characterized by intense microtopographic variation and erratic spatial salinity gradients. As documented in similar complex coastal environments globally, these dynamic abiotic fluctuations interact strongly with species-specific morphological traits (namely shallow rooting depth, low below-ground biomass, and patchy rhizosphere development). Together, these factors drive a highly localized, erratic distribution of organic matter that inherently resists simple, one-dimensional depth modeling. Therefore, rather than viewing this low variance merely as a statistical limitation, it provides a coherent interpretation of a fundamental ecological reality: SOC_*v*_ estimates for functionally distinct, patchily distributed species like *S. fruticosa* must be interpreted with caution. Consequently, for effective management and restoration applications, future research must move beyond simple vertical profiles and employ spatially explicit modeling frameworks that incorporate these critical horizontal environmental predictors (e.g., salinity gradients and texture heterogeneity).

Although all three models produced strong fits (*R²* > 0.997) for SOC_*c*_ depth distribution across the three studied plant species (Table 6), identifying the model with the lowest prediction error is essential for accurately estimating SOC_*c*_. In this study, the sigmoid model provided the best fit for unvegetated sites, *A. macrostachyum*, and *S. fruticosa*, while the allometric model was most effective for *H. strobilaceum*. Comparing these model selections with global and regional literature reveals that the optimal mathematical function is not universally fixed; rather, it is highly dependent on species-specific traits and local vegetation structure [7, 22, 26, 65]. Across various salt marsh ecosystems, the transition between best-fitting models mathematically mirrors distinct vertical carbon dynamics. For instance, the superior fit of the allometric model for *H. strobilaceum* (a pattern also observed for certain deep-rooted halophytes in comparable studies) likely captures power-law scaling associated with its pronounced below-ground biomass and root architecture. Conversely, the widespread effectiveness of the sigmoid model excellently captures the transitional nature of soil profiles, where an organically rich, biologically active surface layer gradually gives way to a stable, compacted mineral subsoil. Therefore, evaluating these modeling approaches collectively demonstrates that they are not merely statistical abstractions, but vital mechanistic tools that translate the unique eco-physical footprints of different plant communities into accurate predictive estimations.

### Limitations and uncertainties

Physico-chemical soil properties were measured exclusively in the 0–20 cm layer because this surface horizon is universally recognized across salt marsh ecosystems as the primary locus of biological activity, dense root networks, active organic matter inputs, and intense biogeochemical cycling [19]. Comparing our sampling framework with broader wetland studies confirms that this upper layer is the most ecologically relevant zone for capturing the dynamic abiotic factors (such as salinity, nutrient availability, and texture) that predominantly dictate surface carbon inputs. Conversely, robustly modeling the vertical attenuation of SOC and accurately calculating SOC_*v*_ and SOC_*c*_ required extending our carbon measurements down to a depth of 50 cm. As demonstrated in various global coastal systems, capturing this extended vertical profile is essential to mathematically define the transition from the biologically active surface to the more stable, mineral-dominated subsoil [22, 26]. While focusing our comprehensive physico-chemical analyses on the biologically critical topsoil optimized analytical resources without compromising the study’s primary objectives, we acknowledge that the lack of continuous deeper physico-chemical profiling remains a limitation. Future studies across comparable systems would benefit from incorporating high-resolution, depth-dependent chemical analyses to further elucidate the complex below-ground drivers of vertical SOC variability.

SOC content was estimated from soil organic matter (SOM) using the conversion equation developed by Maxwell et al. [39], which is based on a global dataset of tidal marsh soils. We verified that the SOM values obtained in this study fall within the lower to mid-range of the calibration dataset reported by Maxwell et al., indicating that the model is applied within its intended parameter space. Nevertheless, we acknowledge that some uncertainty may arise from using a globally derived empirical relationship in a hyper-arid coastal system characterized by high salinity, limited moisture, and predominantly sandy substrates. These environmental differences may influence SOM composition and, consequently, SOC conversion efficiency. Therefore, while the Maxwell equation provides a more robust alternative to fixed conversion coefficients, we recommend future work to directly measure SOC (e.g., via CHN analysis) for a subset of samples to validate conversion accuracy under local conditions.

In the present study, exceptionally high *R²* values (*R²* > 0.997) were achieved for SOC_*c*_, whereas modeling SOC_*v*_ yielded comparatively lower and more variable goodness-of-fit metrics across unvegetated areas and marshes dominated by *A. macrostachyum*, *H. strobilaceum*, and *S. fruticosa*. When evaluated through the lens of broader soil carbon modeling, this statistical divergence is a well-recognized methodological phenomenon rather than a site-specific anomaly. In dynamic salt marsh ecosystems, vertical SOC_*v*_ profiles are inherently subject to intense, localized eco-physical fluctuations (such as root biomass clustering and sporadic sediment deposition) which naturally reduce the variance explained by simple depth-dependent models. Conversely, the near-perfect metrics achieved for SOC_*c*_ are a direct mathematical consequence of progressive cumulative integration from the soil surface downward. Continuously summing positive carbon values layer by layer inherently suppresses this localized biological ‘noise’, forcing a smoothed, consistently increasing mathematical trajectory. Therefore, critically comparing these modeling outcomes provides a coherent interpretation for carbon accounting across coastal wetlands: the exceptionally high *R²* values for SOC_*c*_ successfully validate the models’ capacity to interpolate internal, within-core vertical inventories. However, they establish strict explicit boundaries, confirming that this mathematical smoothing must not be misconstrued as evidence of broad spatial predictability across un-sampled, heterogeneous landscapes.

## Conclusions

This study provides new insights into the vertical distribution and storage of SOC in arid coastal salt-marsh ecosystems by applying depth-response models across multiple vegetation types. Species-specific differences demonstrate that vegetation composition plays a central role in shaping carbon storage potential in these systems. Based on these findings, several policy-relevant recommendations emerge for coastal management and carbon sequestration efforts: First, restoration programs should prioritize the protection and re-establishment of vegetation types that demonstrated strong SOC-sequestration potential and reliable predictive modeling (specifically *H. strobilaceum* and *A. macrostachyum*). In contrast, while *S. fruticosa* is present in these ecosystems, its high spatial variability resulted in poor model performance. Consequently, predictions regarding its sequestration capacity should be interpreted with caution, and it cannot currently be recommended as a reliable priority for management decisions until more robust models are developed. Second, policymakers should focus on conserving the upper soil layers (0–20 cm), where SOC is most concentrated and most vulnerable to disturbance. This includes restricting activities such as uncontrolled grazing, vehicular access, and shoreline modification that degrade topsoil carbon. Third, establishing long-term monitoring and carbon-accounting programs is essential for tracking changes in SOC_*c*_ and evaluating restoration outcomes; the SOC values and model outputs generated here provide critical baseline data for such efforts. Fourth, policymakers should invest in public awareness programs to empower local communities with knowledge about the role of vegetation in carbon sequestration, fostering long-term stewardship of these fragile habitats. Finally, while the baseline data established in this study can inform local restoration initiatives, these predictive models require independent site-level validation before assuming broad spatial applicability. Additionally, targeted investigations are needed to resolve the environmental drivers behind the high spatial variability observed in *S. fruticosa* to develop more reliable carbon accounting tools across diverse coastal environments.

## Supplementary Information

Supplementary Material 1.

Supplementary Material 2.

## Data Availability

The data supporting the findings of this study are available upon reasonable request.
