# Supplementary material for: Modeling the distribution of soil organic carbon in salt marshes dominated by various plant species along Egypt’s Delta coast
Source: BMC Plant Biol. 2026 Jun 17;26:1053. doi: 10.1186/s12870-026-09221-2 (PMC13277068; doi:10.1186/s12870-026-09221-2)
Supplement: Supplementary file 1 — Supplementary Material 1. [file 12870_2026_9221_MOESM1_ESM.docx]

**
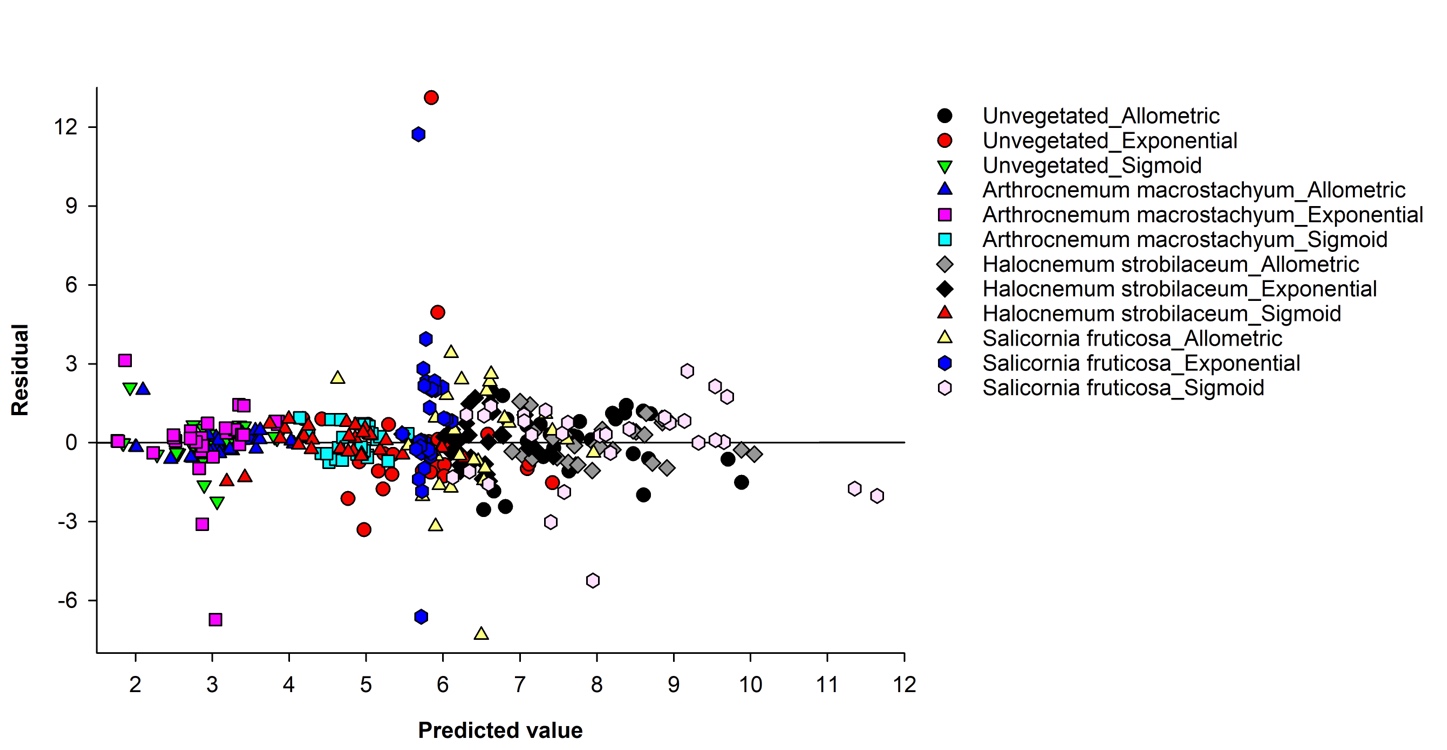
**

**Fig. S1** Model uncertainty (predicted vs. residuals) for volumetric soil organic carbon density (SOC*_v_*; kg C/m³) using three mathematical models across salt marshes with different plant species along the deltaic coast of Egypt.


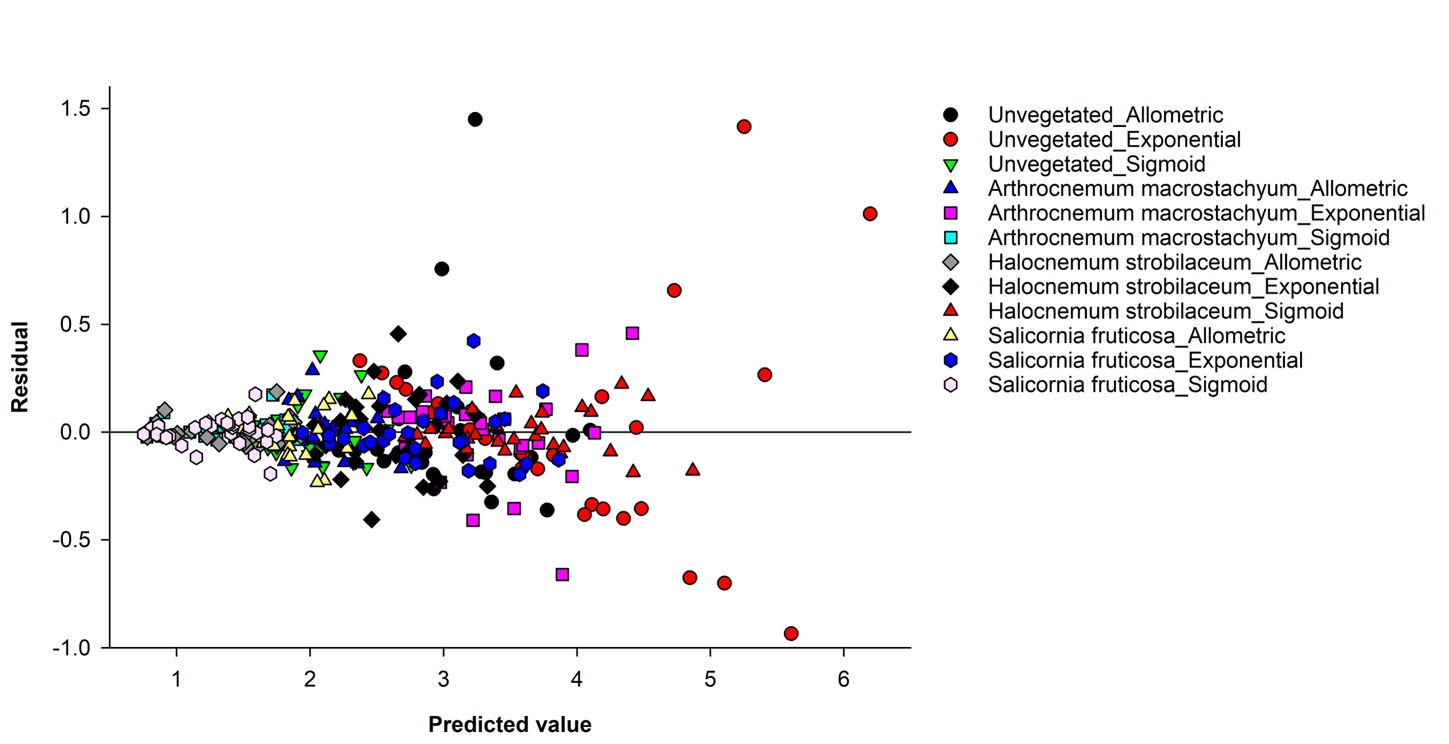


**Fig. S2** Model uncertainty (predicted vs. residuals) for cumulative soil organic carbon stock (SOC*_c_*; kg C/m²) using three mathematical models across salt marshes with different plant species along the deltaic coast of Egypt.
