# Supplementary material for: Modeling the distribution of soil organic carbon in salt marshes dominated by various plant species along Egypt’s Delta coast
Source: BMC Plant Biol. 2026 Jun 17;26:1053. doi: 10.1186/s12870-026-09221-2 (PMC13277068; doi:10.1186/s12870-026-09221-2)
Supplement: Supplementary file 2 — Supplementary Material 2. [file 12870_2026_9221_MOESM2_ESM.docx]

**Table S1** The coordinates of each sampling location in salt marshes dominated by different plant species along Egypt’s deltaic coast

| **Location number** | **Latitude** | **Longitude** | **Species** | **Number of collected cores per species** | **Number of collected cores per location** |
| --- | --- | --- | --- | --- | --- |
| 1 | 31°26'24.32"N | 31°36'0.86"E | *Arthrocnemum macrostachyum*  *Halocnemum strobilaceum*  *Salicornia fruticosa* | 4 | 11 |
|  |  |  |  | 3 |  |
|  |  |  |  | 4 |  |
| 2 | 31°31'3.65"N | 31°20'27.08"E | *Halocnemum strobilaceum*  *Salicornia fruticosa*  Unvegetated | 3 | 10 |
|  |  |  |  | 3 |  |
|  |  |  |  | 4 |  |
| 3 | 31°31'25.06"N | 31°19'49.96"E | *Halocnemum strobilaceum*  *Salicornia fruticosa*  Unvegetated | 3 | 10 |
|  |  |  |  | 3 |  |
|  |  |  |  | 4 |  |
| 4 | 31°29'4.11"N | 31°24'37.22"E | Unvegetated | 6 | 6 |
| 5 | 31°29'1.54"N | 31°24'37.53"E | *Arthrocnemum macrostachyum*  *Salicornia fruticosa*  Unvegetated | 5 | 13 |
|  |  |  |  | 3 |  |
|  |  |  |  | 5 |  |
| 6 | 31°31'59.22"N | 31°19'0.63"E | Unvegetated | 6 | 6 |
| 7 | 31°31'49.32"N | 31°18'55.58"E | Unvegetated | 6 | 6 |
| 8 | 31°26'21.64"N | 30°32'6.88"E | *Arthrocnemum macrostachyum*  *Halocnemum strobilaceum*  *Salicornia fruticosa* | 5 | 11 |
|  |  |  |  | 3 |  |
|  |  |  |  | 3 |  |
| 9 | 31°26'16.54"N | 30°31'42.97"E | *Halocnemum strobilaceum*  *Salicornia fruticosa* | 4 | 9 |
|  |  |  |  | 5 |  |
| 10 | 31°26'33.26"N | 30°32'54.14"E | *Halocnemum strobilaceum*  *Salicornia fruticosa* | 4 | 10 |
|  |  |  |  | 6 |  |
| 11 | 31°27'27.21"N | 30°37'8.21"E | *Arthrocnemum macrostachyum*  *Halocnemum strobilaceum*  Unvegetated | 5 | 13 |
|  |  |  |  | 4 |  |
|  |  |  |  | 4 |  |
| 12 | 31°27'33.54"N | 30°37'8.31"E | *Arthrocnemum macrostachyum*  *Halocnemum strobilaceum*  Unvegetated | 6 | 14 |
|  |  |  |  | 4 |  |
|  |  |  |  | 4 |  |
| 13 | 31°27'56.12"N | 30°39'24.34"E | *Halocnemum strobilaceum* | 4 | 4 |
| 14 | 31°28'17.02"N | 30°38'43.21"E | *Halocnemum strobilaceum*  *Salicornia fruticosa* | 4 | 10 |
|  |  |  |  | 6 |  |
| 15 | 31°30'14.73"N | 30°45'33.26"E | Unvegetated | 6 | 6 |
| 16 | 31°34'12.73"N | 30°56'40.41"E | *Arthrocnemum macrostachyum*  *Halocnemum strobilaceum*  *Salicornia fruticosa*  Unvegetated | 6 | 18 |
|  |  |  |  | 4 |  |
|  |  |  |  | 4 |  |
|  |  |  |  | 4 |  |
| 17 | 31°33'31.51"N | 30°54'22.11"E | *Arthrocnemum macrostachyum*  *Salicornia fruticosa*  Unvegetated | 5 | 12 |
|  |  |  |  | 3 |  |
|  |  |  |  | 4 |  |
| 18 | 31°32'8.33"N | 30°49'49.63"E | *Arthrocnemum macrostachyum*  *Halocnemum strobilaceum* | 6 | 10 |
|  |  |  |  | 4 |  |
| 19 | 31°28'27.95"N | 30°40'55.82"E | *Halocnemum strobilaceum*  *Salicornia fruticosa* | 4 | 9 |
|  |  |  |  | 5 |  |
| 20 | 31°27'8.81"N | 30°35'36.75"E | *Arthrocnemum macrostachyum*  *Halocnemum strobilaceum*  Unvegetated | 5 | 12 |
|  |  |  |  | 3 |  |
|  |  |  |  | 4 |  |

**Table S2** Distribution of sampling locations and collected soil cores across vegetation types in salt marshes along Egypt’s deltaic coast

| **Category / Vegetation Condition** | **Number of Locations** | **Number of Soil Cores Collected** |
| --- | --- | --- |
| **Unvegetated areas** | 12 | 57 |
| ***Arthrocnemum macrostachyum*** | 9 | 47 |
| ***Halocnemum strobilaceum*** | 14 | 51 |
| ***Salicornia fruticosa*** | 11 | 45 |
| **Total** | **20 locations*** | **200 soil cores** |

*The 20 locations include sites that contained one, two, or all three species, and some also included unvegetated patches; therefore, the number of locations across categories exceeds 20 due to overlapping vegetation types within the same site
